# Acupuncture for stroke survivors with cardiovascular comorbidities: protocol of a pooled analysis of randomized controlled trials

**DOI:** 10.3389/fneur.2025.1502091

**Published:** 2025-04-14

**Authors:** Lanfang Zhang, Zubing Mei, Zhenhua Xu

**Affiliations:** ^1^Department of Acupuncture and Rehabilitation Therapy, The Second Affiliated Hospital of Guangzhou University of Chinese Medicine, Guangzhou, China; ^2^The Second Clinical Medical College of Guangzhou University of Chinese Medicine, Guangzhou, China; ^3^Guangdong Provincial Hospital of Traditional Chinese Medicine, Guangzhou, China; ^4^Department of Anorectal Surgery, Shuguang Hospital, Shanghai University of Traditional Chinese Medicine, Shanghai, China; ^5^Department of Acupuncture and Moxibustion, The Second Affiliated Hospital of Guangzhou University of Chinese Medicine, Guangzhou, China

**Keywords:** acupuncture, stroke survivors, cardiovascular comorbidities, rehabilitation, pooled analysis, randomized controlled trials (RCTs), neurological function, quality of life

## Abstract

**Background:**

Stroke remains a leading cause of long-term disability globally, often complicated by cardiovascular comorbidities such as hypertension, atrial fibrillation, and coronary artery disease. These comorbidities not only increase the risk of recurrent strokes but also complicate recovery, making effective rehabilitation strategies crucial. Acupuncture, a traditional Chinese medicine practice, has gained attention as a potential complementary therapy for stroke rehabilitation. This protocol outlines a pooled analysis of randomized controlled trials (RCTs) aimed at evaluating the effectiveness and safety of acupuncture in improving neurological function, quality of life, and reducing stroke recurrence in stroke survivors with cardiovascular comorbidities.

**Methods and analysis:**

This pooled analysis will adhere to the Preferred Reporting Items for Systematic Reviews and Meta-Analyses (PRISMA) guidelines. We will include RCTs that assess acupuncture interventions compared to placebo acupuncture, standard care, or no intervention in adult stroke survivors with at least one cardiovascular comorbidity. Comprehensive literature searches will be conducted in databases including PubMed, Embase, Cochrane Central Register of Controlled Trials (CENTRAL), and CINAHL, covering all studies published from inception to the most recent update. Two independent reviewers will screen titles and abstracts, and full-text articles will be reviewed to confirm eligibility. Data extraction will focus on participant characteristics, intervention specifics, outcomes related to neurological function, quality of life, stroke recurrence, and adverse events. The risk of bias will be assessed using the Cochrane Risk of Bias tool. A random-effects meta-analysis will be conducted to account for heterogeneity across studies, and subgroup analyses will explore the impact of different stroke types, cardiovascular comorbidities, and acupuncture techniques on outcomes.

**Discussion and conclusion:**

This study aims to provide a comprehensive evaluation of the role of acupuncture as an adjunctive treatment for stroke rehabilitation in patients with cardiovascular comorbidities. The findings are expected to contribute to the evidence base regarding the efficacy and safety of acupuncture in this specific patient population, potentially influencing clinical practice guidelines and promoting the integration of acupuncture into standard stroke care protocols. Despite the potential benefits, challenges such as variability in acupuncture techniques and patient characteristics across studies may affect the generalizability of the results. Nevertheless, this study will offer valuable insights into optimizing rehabilitation strategies for stroke survivors with complex clinical profiles.

**PROSPERO registration no:**

CRD42024576156.

## Introduction

Stroke, a leading cause of death and long-term disability globally, imposes a significant burden on healthcare systems, patients, and families alike. The combination of stroke and cardiovascular comorbidities, such as hypertension, atrial fibrillation, and coronary artery disease, complicates the clinical picture, contributing to a higher risk of recurrent stroke, increased mortality, and prolonged recovery periods. Cardiovascular conditions not only increase stroke risk but also exacerbate post-stroke recovery, impairing functional outcomes and diminishing quality of life in survivors (1, 2). In fact, patients with both stroke and cardiovascular comorbidities experience worse prognoses compared to those with stroke alone, as these conditions can lead to systemic inflammation, impaired cerebral perfusion, and increased oxidative stress, all of which hinder recovery (3, 4).

Acupuncture, a traditional Chinese medicine practice, has been increasingly recognized as a potential adjunct therapy in stroke rehabilitation. Emerging evidence suggests that acupuncture may offer neuroprotective effects, enhance cerebral blood flow, and modulate autonomic nervous system functions, potentially addressing both neurological deficits and cardiovascular risk factors in stroke survivors (5, 6). Acupuncture’s role in regulating blood pressure, reducing heart rate variability, and decreasing inflammatory markers makes it a promising intervention for stroke patients with cardiovascular comorbidities (7, 8). While several randomized controlled trials (RCTs) have evaluated acupuncture in stroke rehabilitation, few have specifically focused on its efficacy in this high-risk subgroup (9, 10).

Notably, clinical studies have begun to explore acupuncture’s efficacy at the intersection of stroke and cardiovascular comorbidities. For instance, a systematic review of 16 trials indicated that acupuncture significantly lowers blood pressure in stroke patients, a key cardiovascular risk factor, while improving neurological outcomes (7). Another RCT demonstrated that acupuncture is safe and effective in the subacute phase of ischemic stroke patients with hypertension, suggesting its potential to address both conditions concurrently (11). These findings highlight acupuncture’s unique ability to target the dual burden of neurological and cardiovascular dysfunction, yet comprehensive evidence remains limited, underscoring the need for this pooled analysis.

Acupuncture is believed to exert a range of therapeutic effects that may benefit stroke survivors with cardiovascular comorbidities by addressing both neurological and cardiovascular dysfunctions. The neuroprotective properties of acupuncture are linked to its ability to enhance cerebral perfusion, promote neuroplasticity, and reduce oxidative stress and inflammation, key factors that contribute to recovery following a stroke (12, 13). For patients with cardiovascular comorbidities, acupuncture’s ability to regulate autonomic nervous system function, reduce blood pressure, and modulate heart rate variability is particularly relevant, as these factors are critical for managing hypertension and other cardiovascular risk factors (14, 15). Furthermore, acupuncture may improve cardiovascular health through additional mechanisms, such as reducing systemic inflammation via downregulation of pro-inflammatory cytokines (e.g., TNF-*α*, IL-6), enhancing endothelial function to improve vascular integrity, and mitigating oxidative stress, all of which are implicated in the progression of cardiovascular diseases like atherosclerosis and heart failure (16, 17). Collectively, these mechanisms suggest that acupuncture may offer a unique dual benefit by improving both neurological recovery and cardiovascular health, which is essential for stroke survivors with concurrent cardiovascular diseases (5, 18).

Recent clinical trials and translational studies have explored the potential benefits of Traditional Chinese Medicine (TCM) interventions, particularly acupuncture, for stroke survivors with cardiovascular comorbidities. Several RCTs have demonstrated that acupuncture can significantly improve neurological recovery and cardiovascular health in this patient population. A systematic review analyzing 16 clinical trials found that acupuncture could be a viable treatment option for lowering blood pressure (7). Another randomized trial reported that acupuncture intervention was confirmed safe in the subacute phase for ischemic stroke patients coexisting hypertention or other comorbidities (11).

In addition to clinical trials, translational studies have provided insights into the mechanisms underlying the therapeutic effects of acupuncture. Studies also demonstrate that acupuncture modulates neurovascular coupling and enhances cerebral perfusion in animal models of ischemic stroke, suggesting a potential pathway through which acupuncture exerts its neuroprotective effects (13, 19). Moreover, animal model and biomarker analysis studies showed that acupuncture reduces systemic inflammation and oxidative stress markers (20, 21), which are critical factors in both stroke recovery and cardiovascular disease management.

These findings underscore the growing recognition of acupuncture and related TCM interventions as valuable adjunctive therapies for stroke survivors, particularly those with coexisting cardiovascular comorbidities. As further studies are conducted, the understanding of these therapies’ clinical applications and underlying mechanisms continues to evolve, offering promising avenues for integrative approaches to stroke rehabilitation and cardiovascular management.

Despite the growing body of evidence supporting the efficacy of acupuncture and other TCM interventions in stroke rehabilitation, significant gaps remain regarding the specific benefits of acupuncture for stroke survivors with cardiovascular comorbidities. Much of the existing literature focuses on either stroke recovery or cardiovascular management in isolation, with limited exploration of the intersection between these two critical health challenges. Furthermore, while some studies suggest that acupuncture can improve both neurological outcomes and cardiovascular parameters, the heterogeneity of study designs and outcomes makes it difficult to draw definitive conclusions (7, 11). Thus, a more rigorous and comprehensive evaluation of acupuncture’s role in managing this dual burden of disease is needed to guide clinical practice.

The present study aims to fill this gap by conducting a pooled analysis of RCTs that investigate the effects of acupuncture on stroke survivors with cardiovascular comorbidities. The primary outcome is neurological recovery, while secondary outcomes include cardiovascular risk factors (e.g., blood pressure control, heart rate variability), stroke recurrence, and quality of life measures.

By synthesizing data from high-quality RCTs, this study seeks to provide robust evidence on the efficacy of acupuncture in addressing both stroke recovery and cardiovascular health. The findings have the potential to inform clinical guidelines and promote integrative approaches to managing stroke patients with complex comorbidities. In doing so, this study could bridge critical gaps in the current literature and offer valuable insights into optimizing care for this vulnerable population.

## Methods

### Study registration

The present study will be conducted following the methodological standards outlined in the Cochrane Handbook for Systematic Reviews of Interventions (22). This guideline will direct our review process, data collection, and statistical analysis, ensuring a rigorous and unbiased approach to synthesizing evidence. To enhance transparency and reproducibility, this protocol adheres to the Preferred Reporting Items for Systematic Reviews and Meta-Analysis Protocols (PRISMA-P) guidelines (23). These standards will ensure comprehensive documentation of the research process, including objectives, search strategies, inclusion and exclusion criteria, and outcome measures.

This study protocol has been registered in the International Prospective Register of Systematic Reviews (PROSPERO) to ensure transparency and reproducibility throughout the research process. The registration provides comprehensive details regarding the study’s objectives, inclusion and exclusion criteria, outcome measures, and methods for conducting a pooled analysis of RCTs on acupuncture interventions for stroke survivors with cardiovascular comorbidities. The registration number for this protocol is CRD42024576156. Additionally, the full protocol can be accessed on the PROSPERO platform using the registration number provided,^1^ which ensures the research’s transparency and accountability.

### Study design

This study will adopt a pooled analysis of RCTs to evaluate the effectiveness of acupuncture interventions among stroke survivors with cardiovascular comorbidities. The analysis will incorporate both individual participant data (IPD) and aggregate study-level data, depending on the availability of data from the included trials. This approach enables a comprehensive examination of the impact of acupuncture across different study settings and populations while minimizing confounding variables and biases that may arise from analyzing single trials. The integration of data from multiple RCTs will involve synthesizing both summary statistics and raw patient-level data, enhancing the overall statistical power and precision of the effect estimates. The primary focus will be on studies that specifically investigate the use of acupuncture as an intervention for managing cardiovascular comorbidities in stroke survivors. Each eligible RCT will be rigorously evaluated to ensure comparability regarding intervention protocols, patient demographics, and outcome measures. This meticulous selection process will help ensure that the results generated from this pooled analysis are valid, reliable, and generalizable to the broader population of stroke survivors who also suffer from cardiovascular conditions.

### Data sources

A comprehensive and systematic search strategy will be employed to identify relevant RCTs for inclusion in this pooled analysis. The search will be conducted across several major electronic databases, including PubMed, EMBASE, the Cochrane Central Register of Controlled Trials (CENTRAL), and CINAHL. These databases were chosen due to their extensive coverage of biomedical literature, particularly in fields pertinent to stroke, cardiovascular disease, and complementary therapies mainly including acupuncture. A specialized health sciences librarian will collaborate in formulating an optimal search strategy that encompasses both controlled vocabulary (MeSH terms) and relevant free-text keywords related to acupuncture, stroke, and cardiovascular conditions (Table 1). The search will also extend to clinical trial registries, such as ClinicalTrials.gov and the World Health Organization International Clinical Trials Registry Platform (WHO ICTRP), to capture ongoing and unpublished studies. Additionally, manual searches of reference lists from included studies and relevant systematic reviews will be performed to ensure comprehensive coverage of all pertinent literature. This thorough approach aims to minimize publication bias and ensure that all relevant data are considered in our analysis, thereby maximizing the robustness and applicability of the findings to clinical practice.

**Table 1:** Detailed PubMed search strategy using a combination of MeSH terms and free-text keywords.

Search component	Search terms
Population (Stroke survivors)	(“Stroke”[Mesh] OR “Cerebrovascular Disorders”[Mesh] OR stroke[tiab] OR cerebrovascular accident[tiab] OR cerebrovascular disease*[tiab] OR ischemic stroke[tiab] OR hemorrhagic stroke[tiab]) AND (“Survivors”[Mesh] OR survivor*[tiab] OR patient*[tiab])
Intervention (Acupuncture)	(“Acupuncture Therapy”[Mesh] OR “Acupuncture”[Mesh] OR acupuncture[tiab] OR electroacupuncture[tiab] OR laser acupuncture[tiab] OR “Traditional Chinese Medicine”[Mesh] OR TCM[tiab] OR “manual acupuncture”[tiab])
Comorbidities (Cardiovascular diseases)	(“Cardiovascular Diseases”[Mesh] OR “Hypertension”[Mesh] OR “Atrial Fibrillation”[Mesh] OR “Coronary Artery Disease”[Mesh] OR cardiovascular[tiab] OR heart disease*[tiab] OR heart failure[tiab] OR atrial fibrillation[tiab] OR hypertension[tiab] OR coronary artery disease[tiab])
Outcome (Rehabilitation and related)	(“Rehabilitation”[Mesh] OR rehabilitation[tiab] OR recovery[tiab] OR “Quality of Life”[Mesh] OR quality of life[tiab] OR functional recovery[tiab] OR “Neurological Function”[tiab] OR “stroke recurrence”[tiab] OR recurrence[tiab] OR “Cardiovascular Risk Factors”[tiab] OR blood pressure[tiab] OR heart rate variability[tiab])
Study type (RCTs)	(“Randomized Controlled Trial”[Publication Type] OR “RCT”[tiab] OR “Randomized Controlled Trial”[tiab])

### Eligibility criteria

The selection process will be conducted following the PICOS (Population, Intervention, Comparison, Outcome, Study design) framework to ensure that all included studies meet the required standards for rigorous evaluation. The specific criteria for inclusion are detailed below:

**Population**: The target population for this pooled analysis comprises adult individuals aged 45 years and older who have experienced any form of stroke, including ischemic stroke, hemorrhagic stroke, or transient ischemic attack (TIA). Additionally, to be included, patients must have at least one documented cardiovascular comorbidity, such as hypertension, coronary artery disease, atrial fibrillation, or heart failure. This age spectrum reflects the higher incidence of stroke and cardiovascular comorbidities in middle-aged and elderly populations. The inclusion of diverse stroke types and cardiovascular conditions is critical to allow for a comprehensive analysis of acupuncture’s potential therapeutic effects across a broad spectrum of stroke survivors with varying cardiovascular profiles.

**Intervention**: The intervention under investigation in this analysis is acupuncture, administered in any form, including traditional manual acupuncture, electroacupuncture, or laser acupuncture. Laser acupuncture, a non-invasive modality using low-level laser stimulation, is included to capture a comprehensive range of acupuncture techniques used in clinical practice. Emerging evidence suggests it may improve microcirculation, reduce inflammation, and support neurological recovery, making it relevant to stroke survivors with cardiovascular comorbidities (24). Eligible studies must describe the acupuncture protocol in detail, including the specific acupuncture points used, the frequency and duration of treatment sessions, and the total number of sessions administered. Additionally, the intervention must be delivered by a licensed and trained acupuncture practitioner to ensure the standardization and quality of the treatment. Studies that incorporate acupuncture as a key component of a multimodal intervention will also be considered, provided that the acupuncture component is clearly delineated and its effects can be independently assessed. This ensures that the impact of acupuncture on both stroke-related outcomes and cardiovascular parameters can be accurately evaluated.

**Comparison**: The comparator for this analysis will include any form of standard care, sham acupuncture, or alternative therapies that do not involve acupuncture. Standard care is defined as the usual medical management provided to stroke survivors with cardiovascular comorbidities, which may include pharmacotherapy, lifestyle modifications, physical rehabilitation, or other conventional treatments. Sham acupuncture refers to procedures that mimic real acupuncture but do not involve needle insertion at therapeutic points, serving as a placebo control.

**Outcome**: The primary outcomes for this analysis will focus on stroke-related functional outcomes and cardiovascular health metrics. Functional outcomes will be assessed using validated scales such as the Modified Rankin Scale (mRS), the Barthel Index, or the National Institutes of Health Stroke Scale (NIHSS), while cardiovascular outcomes will include measures of blood pressure (both systolic and diastolic), heart rate variability, and the occurrence of major adverse cardiovascular events (MACE). Secondary outcomes will include measures of health-related quality of life, using tools like the Stroke Impact Scale (SIS) or the EuroQol-5D (EQ-5D), as well as patient-reported outcomes on pain, anxiety, and depression. Additionally, the analysis will consider the safety profile of acupuncture by documenting adverse events and their frequency across studies.

**Study Design**: The analysis will be limited to RCTs to ensure the highest level of evidence is synthesized. Eligible RCTs must be published in peer-reviewed journals, written in English, and provide sufficient data for the extraction of relevant outcome measures. Trials that report individual participant data (IPD) will be prioritized, but aggregate data from well-conducted RCTs will also be included.

**Exclusion Criteria**: Studies will be excluded if they are non-randomized, lack a clear definition of the acupuncture intervention, or do not report on any of the primary or secondary outcomes outlined in this protocol. Additionally, studies where the acupuncture treatment is not the primary intervention or where the role of acupuncture is insufficiently detailed will be excluded to maintain the specificity of the analysis. Trials involving pediatric populations or those that do not explicitly include participants with cardiovascular comorbidities will also be excluded.

### Study selection

The process of study selection for this pooled analysis will involve a systematic multi-step approach, starting with an initial screening of the titles and abstracts. Two independent reviewers will conduct this preliminary screening using the PICOS framework outlined in the eligibility criteria. This stage is designed to eliminate studies that do not meet the basic inclusion criteria, such as non-randomized trials or studies that lack a clear focus on acupuncture for stroke survivors with cardiovascular comorbidities. In cases where the reviewers disagree on the inclusion of a study, disagreements will be resolved through discussion, and if consensus cannot be reached, a third reviewer will be consulted to make a final decision.

Following the initial screening, the full texts of studies deemed potentially relevant will be obtained for a more detailed assessment of their eligibility. Again, two independent reviewers will assess each full-text article, using a standardized data collection form that ensures consistency and completeness in evaluating the study’s population, intervention, comparator, outcomes, and design. The study selection process will be documented in a PRISMA flow diagram, which will illustrate the number of studies included and excluded at each stage, along with the reasons for exclusion.

### Data extraction

Data extraction will be performed using a pre-defined standardized form that has been pilot-tested to ensure its accuracy and appropriateness for the study. Two independent reviewers will carry out the extraction process to minimize the risk of errors and biases. Any discrepancies in the extracted data will be addressed through discussion and mutual consensus, or if necessary, by involving a third reviewer. The reviewers will extract key study characteristics, including publication details (e.g., authors, year of publication, and country of origin), participant demographics (e.g., age, gender, stroke type, and cardiovascular comorbidities), specifics of the acupuncture intervention (e.g., type of acupuncture, points used, duration, and frequency of treatment), and details of the comparator (e.g., sham acupuncture or usual care; Table 2).

**Table 2:** Structured data extraction form to help systematically capture key information from the studies included in the systematic review.

Data category	Data element	Description/notes
Publication details	Authors	List all authors
	Year of Publication	Year when the study was published
	Country of Origin	Country where the study was conducted
Participant demographics	Age	Average age or age range of participants
	Gender	Distribution of gender in the study population
	Stroke Type	Types of stroke included (e.g., ischemic, hemorrhagic)
	Cardiovascular Comorbidities	List of cardiovascular comorbidities (e.g., hypertension, atrial fibrillation)
Acupuncture intervention	Type of Acupuncture	Specify the type (e.g., manual, electroacupuncture, laser)
	Points Used	Acupuncture points used during treatment
	Duration of Treatment	Duration of each session (in minutes)
	Frequency of Treatment	Frequency of treatment sessions (e.g., daily, weekly)
	Total Number of Sessions	Total number of acupuncture sessions over the treatment period
Comparator details	Type of Comparator	Type of control used (e.g., sham acupuncture, usual care, no intervention)
	Description of Comparator	Detailed description of the comparator intervention, if applicable
Outcomes	Primary Outcomes	Primary outcomes measured (e.g., neurological function, quality of life, stroke recurrence)
	Secondary Outcomes	Secondary outcomes measured (e.g., adverse events, cardiovascular risk factors)
Methodological quality	Randomization	Description of randomization method
	Blinding	Information on whether participants, caregivers, and outcome assessors were blinded
	Follow-up	Length of follow-up period and percentage of participants completing the study
	Risk of Bias	Summary of risk of bias assessment using appropriate tools (e.g., Cochrane Risk of Bias tool)

Outcome data to be extracted will include primary outcomes such as functional outcomes assessed via validated scales (e.g., Modified Rankin Scale) and cardiovascular measures (e.g., blood pressure, heart rate variability). Secondary outcomes will include quality of life, adverse events, and recurrence of stroke or cardiovascular events. Study results will be extracted in the form of effect estimates, including measures such as odds ratios, risk ratios, and confidence intervals where applicable. This comprehensive data extraction process will ensure that all relevant information is captured for the pooled analysis, providing a solid foundation for the synthesis of evidence.

### Risk of bias assessment

The risk of bias in the included studies will be evaluated using the Cochrane Collaboration’s Risk of Bias tool (RoB 2.0) for RCTs, which provides a comprehensive framework for assessing potential bias in several critical domains (25), including bias arising from the randomization process, deviations from the intended interventions, missing outcome data, measurement of the outcome, and the selection of the reported results. Each domain will be rated as having a low risk, some concerns, or a high risk of bias. An overall risk of bias judgment for each study will be made based on the assessment across these domains, following the guidelines provided in the Cochrane Handbook for Systematic Reviews of Interventions.

To ensure consistency and objectivity in the assessment, two independent reviewers will perform the risk of bias evaluation. Any disagreements between reviewers will be addressed through discussion and, if unresolved, a third reviewer will be consulted. The results of the risk of bias assessment will be synthesized and presented in a ‘Risk of Bias’ summary figure and table, providing a visual and quantitative representation of the risk of bias across all included studies.

### Grading of evidence

The quality of evidence across the included studies will be appraised using the Grading of Recommendations, Assessment, Development and Evaluations (GRADE) approach, which is widely recognized for its methodological rigor in evidence synthesis (26). Each outcome will be evaluated across five key domains: risk of bias, inconsistency, indirectness, imprecision, and publication bias. The evidence for each outcome will be graded as high, moderate, low, or very low. These grades reflect the degree of confidence in the effect estimates and the extent to which future research might impact these estimates. A high grade indicates a high level of confidence that the estimate reflects the true effect, while a very low grade suggests considerable uncertainty about the effect estimate (Table 3).

**Table 3:** A structured grading of evidence table which applies the GRADE approach to assess the quality of evidence and strength of recommendations (some of the outcomes as examples).

Outcome	Quality of evidence	Consistency	Directness	Precision	Publication bias	Overall GRADE
Neurological function improvement	Initial high/ moderate	No serious/some inconsistency	Direct/indirect	Precise/imprecise	Undetected/suspected	High/moderate/low
Quality of life	Initial high/ moderate	No serious/some inconsistency	Direct/indirect	Precise/imprecise	Undetected/suspected	High/moderate/low
Stroke recurrence reduction	Initial high/ moderate	No serious/some inconsistency	Direct/indirect	Precise/imprecise	Undetected/suspected	High/moderate/low
Cardiovascular risk factor control	Initial high/ moderate	No serious/some inconsistency	Direct/indirect	Precise/imprecise	Undetected/suspected	High/moderate/low
Adverse events	Initial high/ moderate	No serious/some inconsistency	Direct/indirect	Precise/imprecise	Undetected/suspected	High/moderate/low

**Explanation of columns: Quality of evidence**: Starting assumption about the quality of evidence for each outcome (high, moderate, low, very low). **Consistency**: Presence or absence of significant differences in the results across studies. **Directness**: Extent to which the people, interventions, and outcome measures are similar to those of interest. **Precision**: Degree of certainty surrounding an effect estimate across studies. **Publication bias**: Presence of bias detected through graphical or statistical tests. **Overall GRADE**: The final grade assigned after considering all factors, indicating the level of confidence in the effect estimate for each outcome.

The GRADE approach also allows for consideration of other contextual factors, such as the balance between benefits and harms, the values and preferences of patients, and resource implications, which are particularly pertinent to the evaluation of acupuncture for stroke survivors with cardiovascular comorbidities. The results of the GRADE assessment will be summarized in a ‘Summary of Findings’ table, which will provide a clear and accessible overview of the key findings (27).

### Statistical analysis

Data will be synthesized using meta-analytic techniques where applicable for this study. The decision to employ either a fixed-effect or random-effects model will be based on the degree of heterogeneity observed across the included studies. The heterogeneity will be quantitatively assessed using the I^2^ statistic, which measures the percentage of variation across studies due to heterogeneity rather than chance (28). If substantial heterogeneity is detected (I^2^ > 50%), a random-effects model will be used to account for variability between studies. In contrast, a fixed-effect model will be applied if heterogeneity is low (I^2^ < 50%). Additionally, sources of heterogeneity will be explored through subgroup analyses based on predefined clinical or methodological characteristics such as the type of acupuncture intervention, duration of follow-up, and severity of cardiovascular comorbidities. Furthermore, if significant heterogeneity remains unexplained after subgroup analyses, meta-regression will be performed to examine the impact of continuous variables, such as age, baseline stroke severity, and cardiovascular risk profiles, on the overall treatment effect. This exploratory analysis will provide insights into how various factors may modify the treatment effect of acupuncture in this population.

Publication bias, which can distort the results if studies with non-significant findings are underreported, will be evaluated using funnel plots for each outcome when at least 10 studies are available. Asymmetry in the funnel plots will be further investigated using Egger’s regression test, which statistically assesses the likelihood of publication bias (29). Sensitivity analyses will also be conducted to ensure the robustness of our findings. This will involve excluding studies with high risk of bias, performing a leave-one-out analysis to assess the influence of individual studies, and using the trim and fill method to adjust for publication bias when funnel plot asymmetry is detected (30).

Effect estimates for continuous outcomes (e.g., mean differences) and dichotomous outcomes (e.g., risk ratios) will be calculated with their corresponding 95% confidence intervals. Forest plots will be used to visually represent the results of the meta-analysis, showcasing the individual study estimates and the pooled effect size. If meta-analysis is not feasible due to excessive heterogeneity or insufficient data, a narrative synthesis of the findings will be conducted to provide a comprehensive summary of the evidence. All statistical analyses will be performed using Stata software version 12.0 (Stata Corp LP, College Station, TX), with statistical significance set at *p* < 0.05.

### Amendments

This protocol is considered a dynamic document, and modifications may be made to address emerging issues or incorporate new evidence as the review progresses. Any amendments to the protocol will be carefully considered and documented to ensure transparency and methodological rigor throughout the course of the pooled analysis. These modifications may include changes to the eligibility criteria, statistical approaches, or methods of data synthesis, based on the evolving nature of the evidence. Amendments will be communicated clearly in the final report, detailing the reasons for the changes and their potential impact on the findings.

## Discussion

### Expected results

This pooled analysis aims to comprehensively assess the efficacy of acupuncture in stroke survivors with cardiovascular comorbidities. Acupuncture, a well-established modality in complementary and integrative medicine, has demonstrated promise in managing post-stroke symptoms and cardiovascular conditions in smaller trials. By pooling the results of existing RCTs, we expect to provide robust evidence on whether acupuncture can effectively reduce stroke-related complications, improve cardiovascular function, and enhance overall quality of life for stroke survivors.

Given that hypertension is the most prevalent cardiovascular comorbidity among stroke patients (affecting 70–80% of cases), it is likely that the included population will predominantly feature individuals with this condition, alongside other comorbidities such as atrial fibrillation or coronary artery disease. This reflects real-world epidemiology and underscores the clinical relevance of our findings. To ensure a comprehensive analysis, subgroup analyses will examine acupuncture’s effects across specific comorbidities, including hypertension, allowing us to determine whether its benefits are consistent or vary by condition. This approach ensures that the predominant hypertensive population is fully analyzed within our research direction while maintaining a broad evaluation of cardiovascular comorbidities.

Given the potential mechanisms by which acupuncture may exert beneficial effects—such as enhancing blood circulation, reducing inflammation, and modulating autonomic nervous system responses (14, 18, 31)—it is anticipated that acupuncture may significantly impact cardiovascular risk factors like blood pressure, lipid profiles, and heart rate variability (32). Additionally, improvements in stroke-related functional outcomes, including motor recovery, balance, and cognitive function, are expected based on previous studies in stroke rehabilitation (33, 34). Moreover, acupuncture has been associated with improvements in mental health, particularly anxiety and depression (35, 36), which are prevalent among stroke survivors with cardiovascular comorbidities. Therefore, we anticipate that the pooled analysis will demonstrate a broader range of benefits beyond physical health, potentially contributing to enhanced psychological well-being and improved health-related quality of life.

However, the variability in acupuncture protocols, including frequency, duration, and the specific acupoints used across different studies, may result in heterogeneous outcomes. We expect to observe moderate heterogeneity in the results due to differences in patient populations, stroke severity, and the presence of other cardiovascular risk factors. Nevertheless, the use of meta-regression and subgroup analyses will allow us to explore these variations in detail, potentially providing insights into which patient subgroups or acupuncture protocols are most effective.

### Potential implications

The findings of this pooled analysis have the potential to significantly impact clinical practice and stroke rehabilitation protocols. If acupuncture is found to be effective in improving cardiovascular outcomes and reducing stroke-related complications, it could be integrated into existing stroke management guidelines, particularly for patients with comorbid cardiovascular conditions. This would provide healthcare professionals with an additional non-pharmacological treatment option that may complement conventional therapies, such as antihypertensive and antiplatelet medications. Furthermore, acupuncture’s low cost and favorable safety profile may make it an attractive option for healthcare systems looking to provide cost-effective care to stroke survivors. In settings where access to specialized stroke rehabilitation services is limited, acupuncture could be implemented as a scalable, community-based intervention, potentially improving health outcomes in underserved populations.

From a policy perspective, the validation of acupuncture as a viable treatment modality for stroke survivors with cardiovascular comorbidities could prompt revisions to national and international stroke management guidelines. Additionally, healthcare policymakers may consider funding research and training programs to increase the availability of qualified acupuncture practitioners in stroke rehabilitation settings. This could lead to the development of multidisciplinary care models that integrate acupuncture with conventional rehabilitation therapies, thereby providing holistic care that addresses both physical and cardiovascular health.

The potential for acupuncture to alleviate stroke-related complications and improve cardiovascular health may also have implications for long-term care planning. Stroke survivors with comorbid conditions often require ongoing management to prevent recurrent events and manage chronic cardiovascular issues. If acupuncture is shown to improve these outcomes, it could reduce the burden on healthcare systems by decreasing the frequency of hospital readmissions, reducing the need for intensive medical interventions, and ultimately improving patients’ long-term prognosis.

In addition to clinical and policy implications, the results of this analysis may inform future research. Identifying gaps in the current evidence base, such as the need for more standardized acupuncture protocols or the exploration of long-term outcomes, could guide future clinical trials. Additionally, further research could focus on understanding the underlying mechanisms of acupuncture in stroke rehabilitation, potentially leading to the development of optimized treatment protocols that maximize both neurological recovery and cardiovascular health.

In summary, the results of this study are expected to provide valuable insights into the role of acupuncture in managing the dual challenges of stroke rehabilitation and cardiovascular comorbidities. If successful, this analysis could pave the way for broader acceptance and integration of acupuncture into stroke care, ultimately improving the quality of life and clinical outcomes for stroke survivors.

### Strengths

This protocol presents several significant strengths that ensure the robustness and scientific rigor of the planned pooled analysis. Firstly, by exclusively focusing on RCTs, the highest standard of evidence in clinical research, this study will enhance the reliability of its findings regarding acupuncture’s effects on stroke survivors with cardiovascular comorbidities. The comprehensive search strategy, encompassing various databases and gray literature sources, minimizes the risk of missing pertinent studies and ensures a broad representation of available evidence. Furthermore, the inclusion of both primary and secondary outcomes, such as cardiovascular health, stroke recurrence, functional recovery, and quality of life, will offer a multifaceted evaluation of the intervention’s efficacy. These diverse outcomes will allow for a thorough understanding of how acupuncture affects multiple dimensions of stroke rehabilitation and comorbidity management. Another strength lies in the planned use of meta-regression and subgroup analyses to explore potential sources of heterogeneity, such as differences in acupuncture protocols, stroke severity, and comorbidity profiles. By investigating these factors, the study can provide more tailored insights into which subgroups of patients may benefit most from acupuncture. The rigorous assessment of study quality and the use of sensitivity analyses will further enhance the robustness of the findings. This methodological approach ensures that any biases inherent in individual studies are identified and their potential impact on the pooled results is minimized.

## Limitations

Despite the strengths, several potential limitations must be acknowledged. First, the heterogeneity among included studies may pose a challenge in synthesizing the results. Differences in acupuncture protocols (e.g., manual vs. electroacupuncture, acupoint selection), frequency, duration of interventions, and patient populations (e.g., stroke severity, comorbidity profiles) could lead to variability in outcomes, complicating the interpretation of the pooled data. Although meta-regression and subgroup analyses will be conducted to explore the sources of heterogeneity, unexplained variability may still affect the generalizability of the findings. Second, the risk of publication bias is a concern, wherein studies with positive results are more likely to be published than those with negative or null findings. While efforts will be made to minimize this bias by including unpublished studies and trial registries, it remains a potential threat to the validity of the analysis. Third, the quality of the included studies may vary, with some trials potentially exhibiting high risk of bias (e.g., inadequate blinding or randomization), which could influence the overall results. Sensitivity analyses will be conducted to mitigate this risk, but the findings may still be influenced by the quality of the underlying evidence.

Additionally, the availability of long-term outcome data may be limited, as many RCTs focus on short- to medium-term effects of acupuncture (e.g., 3–6 months post-intervention). This could restrict our ability to assess acupuncture’s impact on stroke recurrence or sustained cardiovascular health improvements, key outcomes for this population. Finally, the lack of standardization in acupuncture protocols across studies may limit comparability, as variations in practitioner expertise, session frequency, and treatment duration could differentially affect outcomes. Combined with the reliance on published data, this may also restrict the level of detail available for individual participants, limiting granular analyses of how patient characteristics (e.g., age, gender, baseline cardiovascular health) modify acupuncture’s effects.

## Ethics and dissemination

As this study is based on a pooled analysis of previously published RCTs, it does not involve direct interaction with human participants. Consequently, the ethical concerns typically associated with clinical trials, such as informed consent and the protection of patient privacy, are not applicable. However, the researchers will ensure that all included studies have been conducted in accordance with ethical standards, including approval by relevant ethics committees and adherence to the Declaration of Helsinki.

The results of this pooled analysis will be disseminated through peer-reviewed publications in high-impact journals, ensuring that the findings reach the broader medical and scientific community. In addition, the results will be presented at national and international conferences focused on stroke rehabilitation, cardiovascular health, and integrative medicine. This dissemination strategy aims to inform clinical practice and stimulate further research in the field of acupuncture for stroke rehabilitation. Moreover, the findings will be shared with healthcare providers and policymakers, with the potential to influence clinical guidelines and healthcare policies related to stroke rehabilitation and the management of cardiovascular comorbidities.

## Conclusion

This study will provide critical insights into the efficacy of acupuncture as an adjunctive therapy for stroke survivors with cardiovascular comorbidities. By synthesizing data from high-quality RCTs, the study will offer robust evidence on the potential benefits of acupuncture in improving cardiovascular health, reducing stroke-related complications, and enhancing overall quality of life. Despite the challenges of heterogeneity and publication bias, the rigorous methodological approach will ensure the validity and reliability of the findings. Should acupuncture prove to be effective, the results could inform clinical guidelines and healthcare policies, advocating for the integration of acupuncture into routine stroke rehabilitation programs, particularly for patients with cardiovascular comorbidities. Moreover, the study’s findings may encourage further research into the mechanisms by which acupuncture exerts its effects, potentially leading to optimized treatment protocols that maximize patient outcomes.

## Footnotes

^1^ https://www.crd.york.ac.uk/prospero/display_record.php?RecordID=576156
